# Art through the Colors of Graffiti: From the Perspective of the Chromatic Structure

**DOI:** 10.3390/s20092531

**Published:** 2020-04-29

**Authors:** Claudia Feitosa-Santana, Carlo M. Gaddi, Andreia E. Gomes, Sérgio M. C. Nascimento

**Affiliations:** 1 Neuroscience for Human Development, Rua Dr Homem de Melo, 697/5154, Sao Paulo 05007-001, Brazil; 2 Experimental Psychology Department, University of Sao Paulo, Sao Paulo 05508-220, Brazil; carlogaddi@usp.br; 3 Centre of Physics, Gualtar Campus, University of Minho, 4710-057 Braga, Portugal; andreia.gomes.ni@gmail.com (A.E.G.); smcn@fisica.uminho.pt (S.M.C.N.)

**Keywords:** color aesthetics, color statistics, color calibration, cultural heritage and art, graffiti, spectral imaging, street art

## Abstract

Graffiti is a general term that describes inscriptions on a wall, a practice with ancient origins, ranging from simple drawings and writings to elaborate pictorial representations. Nowadays, the term graffiti commonly describes the street art dedicated to wall paintings, which raises complex questions, including sociological, legal, political and aesthetic issues. Here we examine the aesthetics of graffiti colors by quantitatively characterizing and comparing their chromatic structure to that of traditional paintings in museums and natural scenes obtained by hyperspectral imaging. Two hundred twenty-eight photos of graffiti were taken in the city of São Paulo, Brazil. The colors of graffiti were represented in a color space and characterized by several statistical parameters. We found that graffiti have chromatic structures similar to those of traditional paintings, namely their preferred colors, distribution, and balance. In particular, they have color gamuts with the same degree of elongation, revealing a tendency for combining similar colors in the same proportions. Like more traditional artists, the preferred colors are close to the yellow–blue axis of color space, suggesting that graffiti artists’ color choices also mimic those of the natural world. Even so, graffiti tend to have larger color gamuts due to the availability of a new generation of synthetic pigments, resulting in a greater freedom in color choice. A complementary analysis of graffiti from other countries supports the global generalization of these findings. By sharing their color structures with those of paintings, graffiti contribute to bringing art to the cities.

## 1. Introduction

Graffiti is a mass noun generally used to describe writings or drawings made on walls to be publicly seen, from the ancient inscriptions to the social phenomenon of tagging names in public locations [1,2]. The term has become even more elastic and in recent decades has also been used to refer to a contemporary form of visual art, usually wall paintings, done illegally or commissioned, in public spaces [3]. There is no consensus in the terminology that describes this more art-related meaning, and this type of graffiti is often called graffiti art [4], independent public art [5], or the most known and general term, street art [6]. In this study, we are concerned with the art expression, and the term graffiti will refer to paintings on public walls, either unsanctioned as in some definitions [1] or sanctioned, as many are nowadays [3].

Whether graffiti are art or visual pollution is still debatable [6]. Negative views relate in general to tagging on public locations [7,8]. Positive views relate to their role in bringing art to the urban environment [6,9,10,11,12] and wellbeing to its habitants [13,14,15,16]. For the scholars, graffiti is considered a new kind of cultural production [11,17] which extrapolates the boundaries of traditional art—“the white cube of the museum”—and makes the city—“the gray cube of the street”—a canvas in an open museum [18]. Moreover, the money involved with the increasing number of famous graffiti artists—e.g., the Banksy canvas “Devolved Parliament” was auctioned for approximately 12 million USD on 3 October, 2019 [19]—and the legal debate about whether the land owner or the graffiti artist owns the graffiti [20,21] clearly express the relevance of graffiti. In any case, its study is now legitimated as a research subject among scholars in the academia [3,22] with dedicated scientific journals such as the *Street Art & Urban Creativity* founded in 2015 [23].

Art, of course, cannot be simply quantified, as it is a complex multidimensional experience [24,25]. It is possible, however, to measure certain properties of artworks, such as the spatial structure [26,27,28,29] and the color structure [30,31,32], and to look for regularities shared by artworks.

The goal of this study was to quantitatively characterize the colors of graffiti and to compare their color structure to that of traditional paintings that are found in museums. We photographed a representative sample of graffiti in different regions of São Paulo, Brazil, with colorimetric precision and analyzed several properties of their color structure. We compared this analysis with a similar analysis of traditional paintings. We found evidence that graffiti and traditional paintings have several color properties in common, suggesting that graffiti represent what is considered art and, therefore, bring art to the cities.

## 2. Material and Methods

### 2.1. Study Area and Image Acquisition

**Figure 1:**
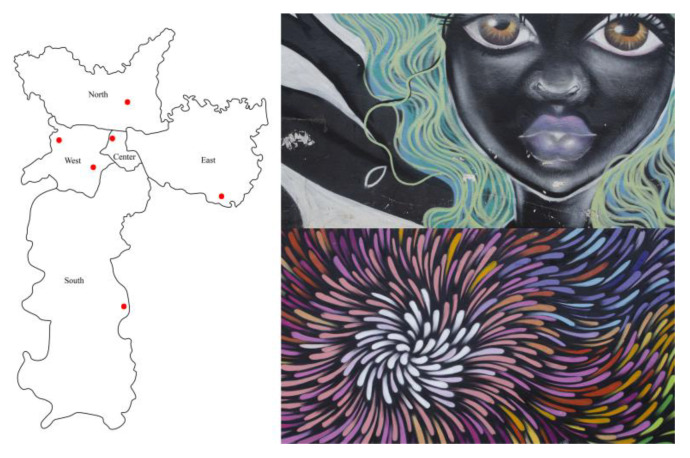
**Left**: red dots indicate the areas within the five zones of the city of São Paulo where the photos of the graffiti were taken. In total, 228 photos were analyzed in the study: 26 from the north, 59 from the south, 44 from the east, 44 from the west, and 55 from the center. The areas were chosen to represent the diversity of styles and selected under the advice of local recognized graffiti artists. **Right**: two examples of graffiti in our sample.

Photos of 245 graffiti of the city of São Paulo, Brazil, were taken in five different zones of the city. Figure 1 shows the locations of these zones on a map of the city as well as some examples of graffiti. From this sample, 17 photos were excluded because of the impossibility to apply the colorimetric calibration due to the non-uniform illumination. Thus, a total of 228 graffiti were analyzed in this study: 26 from the north, 59 from the south, 44 from the east, 44 from the west, and 55 from the center. These photos were taken during the month of October around the middle of the day in order to avoid shade as much as possible. The areas were chosen to represent the diversity of styles, following the advice of local recognized graffiti artists. In each area, the graffiti were photographed in the exact sequence in which they were present in each alley, avoiding subjective aesthetics judgements from the authors of this work. Monochromatic or unevenly illuminated graffiti were not considered.

**Figure 2:**
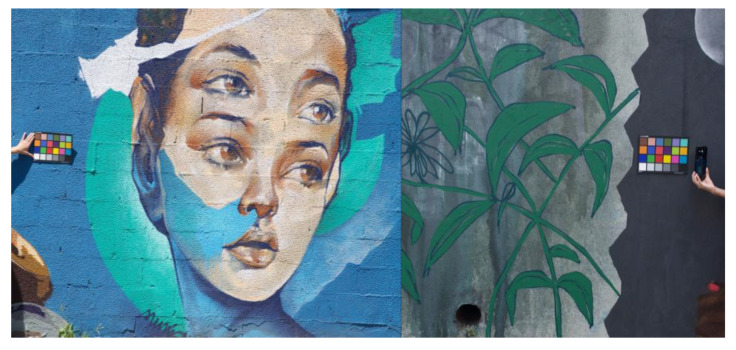
Illustration of the procedure of image acquisition. **Left**: acquiring the image with the X-Rite Macbeth ColorChecker Classic included in the field of view of the photo for colorimetric calibration and spatial scaling purposes. **Right**: acquisition of the illumination spectrum on the color chart with a portable spectro-colorimeter Everfine SPIC-200 for colorimetric calibration (the moment right before the photo).

The camera was a Nikon D7000 with a resolution of 4928 × 3264 pixels, and the optical conditions were such that the average resolution of the system was about 0.5 mm per pixel. An X-Rite Macbeth ColorChecker Classic with 24 samples was included in the field of view of each photo as shown in Figure 2. The ColorChecker was used for calibration purposes, for both colorimetric calibration and spatial scaling. For colorimetric calibration, the spectral irradiance on the color chart was measured immediately before taking the photo with a portable spectro-colorimeter Everfine SPIC-200, in the spectral range from 380 to 760 nm.

### 2.2. Colorimetric Calibration of the Photos

Images were converted from raw camera NEF format to DNG and then converted to sRGB (standard RGB). For the colorimetric calibration, the spectral reflectance of the samples of the color checker were measured in the laboratory with a spectrophotometer Minolta CM 2600d (Konica Minolta Co. Lda., Japan) and used to obtain the exact colors of the patches of the color checker under each local illumination measure at the time of image acquisition for each photo. These data were then used to correct the sRGB data from the camera for each photo using a polynomial regression with least squares fitting with a three-term polynomial [33,34]. The average color error across all 228 photos of the color chart was 6.3 ΔE units before the colorimetric correction. This value was obtained by computing the real colors of the 24 samples of the color chart and comparing with the ones obtained from the camera. After the colorimetric correction, this value was reduced to 4.0 ΔE units, a number that is just a little above the visual limit of 2.2 ΔE to perceive color changes in complex images [35,36].

**Figure 3:**
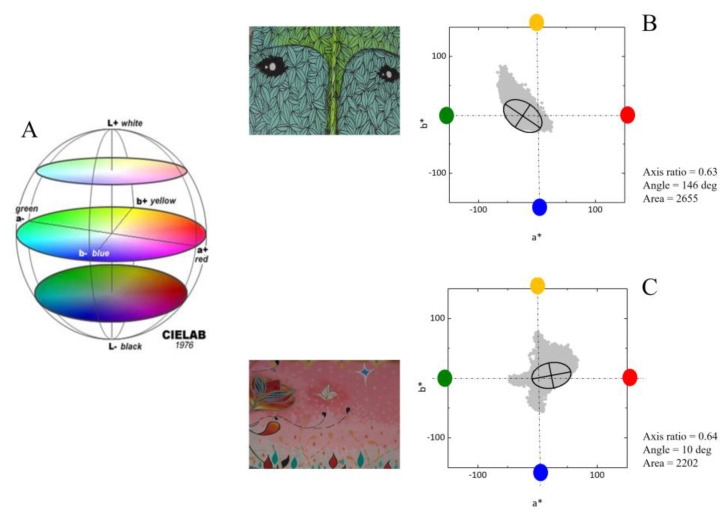
(**A**) The color space CIELAB. (**B**,**C**) The representation of the color gamut of two examples of graffiti in the color plane of the color space. The color of each direction—red, green, yellow and blue—is indicated in the graphs. The upper graffiti (**B**) has more greens and yellows, and the lower graffiti (**C**) has more reds, properties that define how the color gamut is represented in the color space. To characterize the geometry of the color gamut with few parameters we fitted ellipses to the gamut such that the major and minor axes were aligned with the principal directions. The axes sizes were twice the standard deviation. The axis ratio was the result of the minor divided by the major axis. The angle was defined between the major axis and the positive a* axis. The area is proportional to the number of colors in the graffiti.

The corrected colors of each pixel were represented in the CIELAB color space [37,38]. We selected this color space because it is a perceptual space, i.e., it represents perceptions rather than physical variables, and because it is reasonably uniform in the sense that has the same metric across all space. Figure 3A illustrates the three dimensions of this color space: L*, lightness; a*, red–green dimension; and b*, yellow–blue dimension. Figure 3B,C illustrates the representation of the color gamut of two graffiti represented in the (a*, b*) plane of the color space. In this work we were not concerned with lightness, the L* component, but were solely concerned with the chromatic dimensions defined in the plane (a*, b*).

To characterize the structure and geometry of the color gamut with few parameters we computed the directions of the principal components [39,40] and, for visualization purposes, represented ellipses such that the major and minor axes were aligned with these directions and were twice the corresponding standard deviations. These ellipses capture the variability of the colors across the two principal directions in the color space. They take into account not only the extent of the gamut but also the distribution of the colors within the gamut. On average, across all graffiti, these ellipses contain about 80% of all colors of the gamut as a consequence of the fact that the axes are twice the standard deviations. Figure 3B,C illustrates the ellipses corresponding to the gamuts of two graffiti. Note that the directions of the principal components reflect both the extent of the colors in the color plane and their frequency of occurrence. The degree of elongation of the ellipse can be quantified by the axis ratio, the ratio of the minor axis to the major axis, and captures the balance between the colors in the gamut. In the examples shown, axis ratios are around 0.6, showing a moderate degree of elongation. The range of more abundant colors can be captured by the angle of the major axis with the positive a* axis. A red–green variation is expressed by angles around 180° (or 0°), and a yellow–blue variation is expressed by angles around 90° (or 270°).

### 2.3. Comparison with Traditional Paintings

The data obtained from the analysis of graffiti were compared with similar data obtained from hyperspectral imaging of paintings. The 44 paintings were from several epochs, from the Renaissance to the mid 20th century. They encompassed several styles, from figurative to abstract, and were from both famous and anonymous artists. They were digitalized by a hyperspectral imaging system in the spectral range 400 to 720 nm with a resolution of 10 nm. The spatial resolution was 1344 × 1024 pixels and the conditions of acquisition produced a resolution about 0.3 min visual angle per pixel, close to that of the human eye. Details about the paintings and digitalization methodology are described elsewhere [30,31,41]. The spectral reflectance of each pixel was estimated, and the corresponding spectral radiance was obtained assuming the standard illuminant D65. The corresponding color was then computed and expressed in the CIELAB color space following the same procedure as for graffiti.

### 2.4. Spatial Calibration

The colors perceived by an observer viewing a graffiti or a painting depend on the distance of observation. For small distances, the eye can resolve the details and see their colors clearly. For large distances, the eye cannot resolve the spatial details so well, causing the colors to mix additively and less colors to be perceived. The colors that are computed here are valid up to the distance where the visual system stops resolving the individual pixels of the images: about 1.5 m for graffiti and 1.8 m for paintings. These distances correspond to the distance where the angular sizes of the pixels match 1 min of arc, the normal visual acuity.

## 3. Results

**Figure 4:**
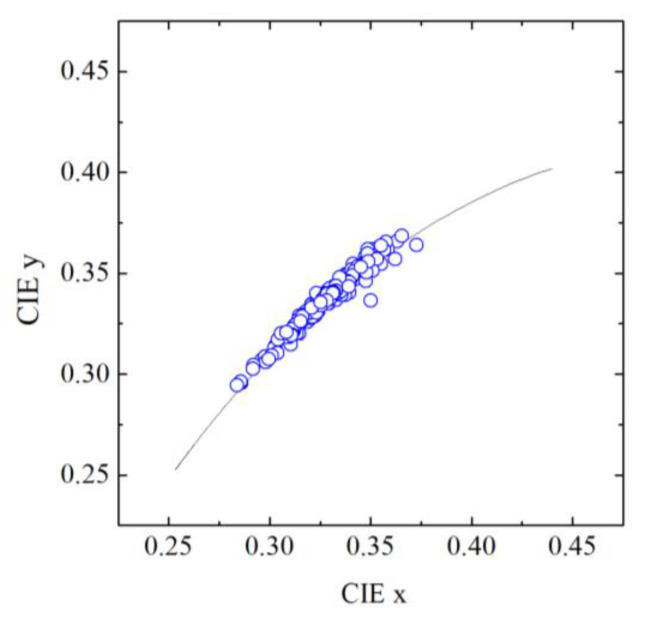
Color of the illumination measured when taking the photos of graffiti (blue symbols). The black line represents the Planckian locus, i.e., the colors of a blackbody with different temperatures, from deep red at low temperatures to bluish white at very high temperatures. The colors of the illumination on graffiti span a considerable range of color temperature corresponding roughly from 5000 K to 10,000 K, expressing different mixtures of skylight and sunlight.

The colors of daylight illumination when taking the photos of graffiti are represented in Figure 4 in the chromaticity diagram 1931 CIE (x, y) by blue symbols. The line represents the Planckian locus, i.e., the colors of a blackbody with different temperatures, from deep red at low temperatures to bluish white at very high temperatures. The colors of the illumination on graffiti span a considerable range of color temperature corresponding roughly from 5000 K to 10,000 K, expressing different mixtures of skylight and sunlight. The colors of graffiti were computed assuming the illuminants at the time of photos. The colors of traditional paintings and pigments considered in the text below were computed assuming the standard illuminant D_65_, but computations with the illuminants obtained from the graffiti are not shown as they did not show any important change.

**Figure 5:**
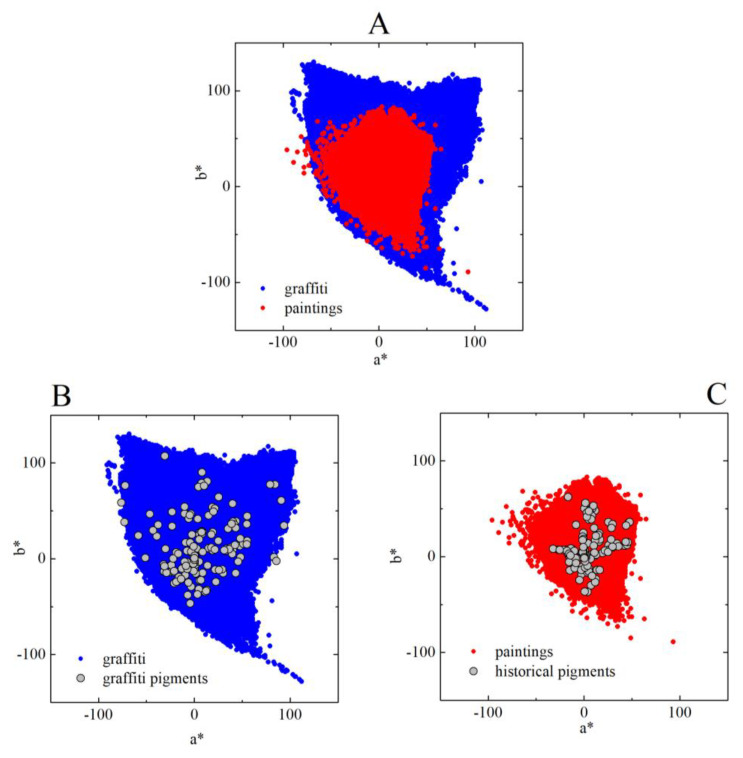
(**A**) Full color gamut obtained from the analysis of 228 graffiti (blue) and 44 paintings (red). (**B**) Gray circles in represent the colors of 150 samples of pigments used in graffiti. (**C**) Gray circles represent the colors of 54 historical pigments commonly used in paintings. The colors of the both types of pigments were obtained assuming standard illuminant D_65_. Graffiti show more saturated colors due to the new generation of artificial pigments.

Figure 5 shows the color gamut obtained from the analysis of the 228 graffiti (blue) and the 44 paintings (red). The gamut of graffiti is considerably larger than that of paintings, showing more intense or saturated colors. To understand the reason for these differences, we collected 150 color samples of pigments used in graffiti and we compared them to the color samples of 54 historical pigments commonly used in paintings retrieved from a database [42]. The color samples of the pigments used for graffiti were obtained by painting small pieces of cardboard with the paint directly from the tin or spray being used by the artists in the streets. These samples were then digitalized by hyperspectral imaging in the laboratory in the same way as the traditional paintings (see Section 2), and their spectral properties were estimated. The spectral properties of the historical pigments used in paintings were obtained with fiber optics reflectance spectroscopy (FORS), a precise spectral technique used in the field of cultural heritage.

Figure 5B shows the colors of graffiti paints, and Figure 5C shows the colors of historical pigments. These colors were computed assuming standard illuminant D65 (as mentioned above, the colors were computed assuming the illuminants measures at the time of image acquisition of graffiti were similar because the CIELAB color space is lowly sensitive to changes in illumination, just like the human eye). Graffiti pigments can be more saturated than historical pigments, explaining the differences in gamut sizes. They are made with contemporary artificial pigments that can be more saturated than those used in our sample of paintings, which ends in the mid 20th century. The palette of contemporary pigments includes new synthetic pigments which can be more permanent and saturated [43]; therefore, if we were analyzing contemporary paintings the gamut sizes obtained would certainly be larger.

**Figure 6:**
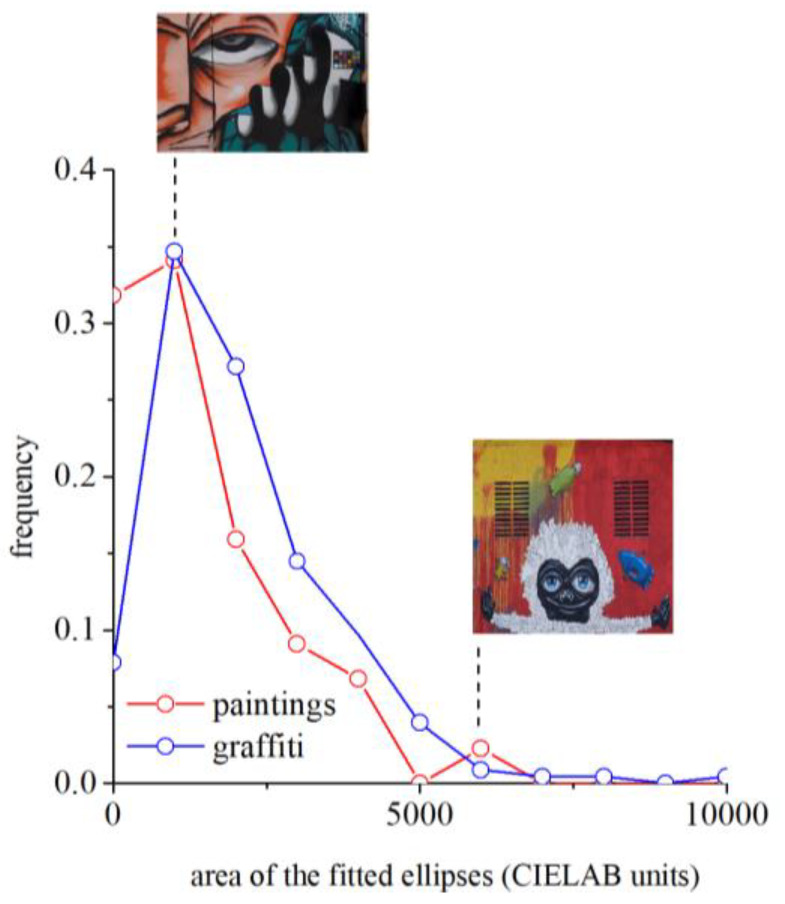
Distribution of the areas of gamuts of the 228 graffiti and 44 paintings expressed in CIELAB units. These areas are a measure of the number of colors that can be seen in each graffiti or painting. As illustration, two examples are shown for the graffiti: one with small area showing a reduced number of colors and one with a large area showing a considerable diversity of colors. The average areas are 6.2 × 10^3^ for graffiti and 3.4 × 10^3^ for paintings. These calculations apply for viewing distance close to the resolution of the human eye, e.g., of about 1.5 m for the graffiti and about 1.8 m for paintings. Graffiti represent more colors than paintings, which translate in the perception of greater chromatic richness.

To look at the gamut of individual graffiti and paintings, the size of the color gamuts, i.e., the area occupied by the colors in the color space, was also computed. Figure 6 represents the distribution of these areas. These areas are a measure of the number of colors that can be seen in each graffiti or painting at viewing distances close to the resolution of the human eye, e.g., of about 1.5 m for the graffiti and about 1.8 m for paintings (see Section 2). For illustration purposes, two examples are shown for the graffiti, one with a small area showing a reduced number of colors and one with a large area showing a considerably greater diversity of colors. The average areas are 6.2 × 10^3^ for graffiti and 3.4 × 10^3^ for paintings. Thus, graffiti represent considerably more colors, which translate into the perception of greater chromatic richness.

**Figure 7:**
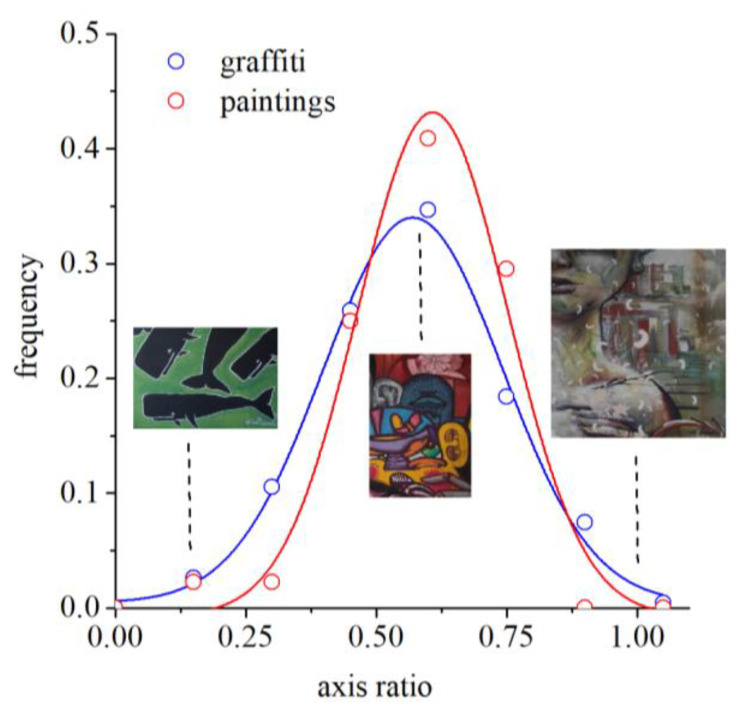
Distributions of axis ratios for graffiti and paintings. This quantity captures the elongation of the color gamut and is obtained from the ratio of the principal directions of variability. Circles represent data points, and lines represent Gaussian fits to the data. The maximum of the Gaussian distribution for graffiti is 0.57 and for paintings is 0.61. As illustration, three examples are shown for the graffiti. The degree of elongation of the gamut is very similar for graffiti and paintings.

The variability of colors across the gamut was quantified by the axis aligned along the principal directions of the colors of the gamut. The axes sizes were proportional to the standard deviation. The axis ratio between minor and major axes gives information about how elongated the gamut is. Thus, values close to one represent gamuts uniformly spread in the color space, whereas values close to zero represent gamuts elongated in a specific direction. Figure 7 shows the distributions of axis ratios for graffiti and paintings. Circles represent data points, and lines represent Gaussian fits to the data. Both samples can be well-described by Gaussians that have maxima in similar values, 0.57 for graffiti and 0.61 for paintings. Thus, graffiti and paintings have gamuts with similar elongations, which translates into similar perceptual balance across perpendicular directions in color space.

**Figure 8:**
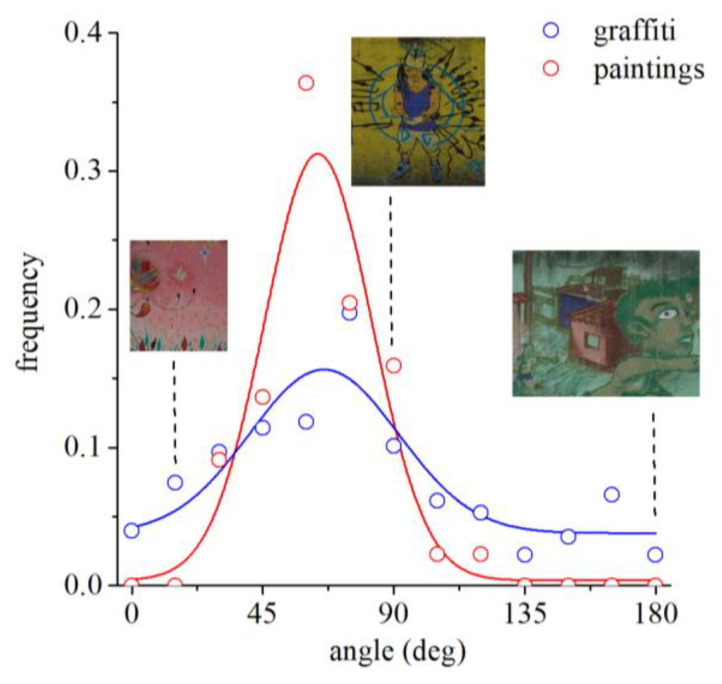
Distributions of the angle of the major axis (first principal direction) with the positive a* axis for graffiti and paintings. This quantity captures the type of colors that show greater variability. Circles represent data points, and lines represent Gaussian fits to the data. The maximum of the Gaussian distribution for graffiti is 66° and for paintings is 64°. As illustration, three examples are shown for the graffiti. The greater variability is in a yellow–blue direction of color space both for graffiti and paintings.

The orientation of the gamut in the color space was characterized by the angle of the major axis with the positive a* axis (see Figure 3 for examples). Figure 8 shows the distributions of these angles for graffiti and paintings. This quantity captures the type of colors that shows greater variability. Circles represent data points and lines represent Gaussian fits to the data. The maximum of the Gaussian distribution for graffiti is 66° and for paintings is 64°. The greater variability is in a yellow–blue direction of color space both for graffiti and paintings. For illustration purposes, three examples are shown for the graffiti. The spread of the distribution of angles for graffiti is wider than for paintings, suggesting more freedom in the use of colors.

**Figure 9:**
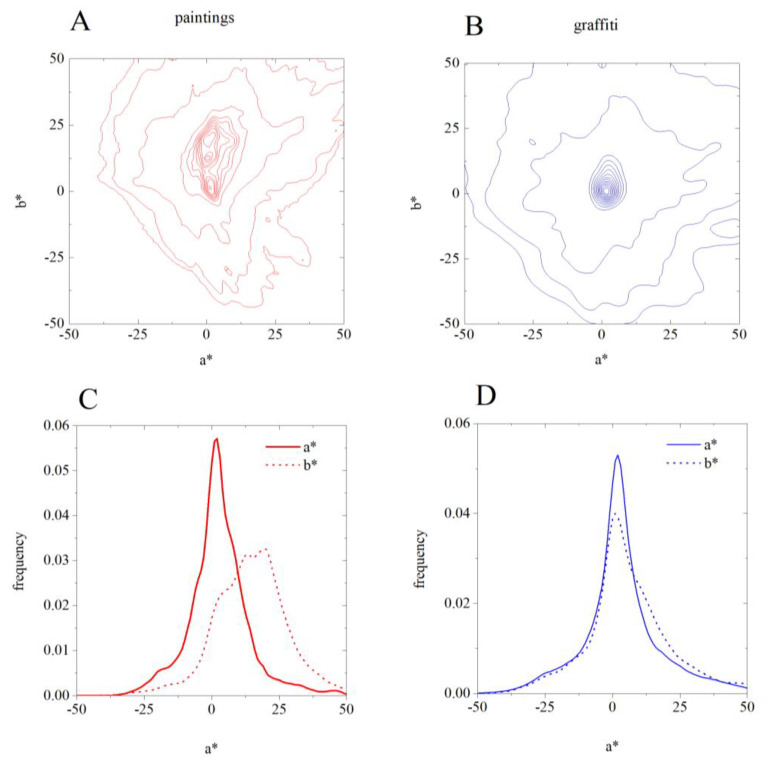
Distribution of the colors in paintings (**A**,**C**) and graffiti (**B**,**D**). Upper panel: contour lines of the frequency of occurrence of the colors in paintings (**A**) and graffiti (**B**). Lower panel: histograms for a* and b* for paintings (**C**) and graffiti (**D**).

The frequency of occurrence of each color was computed to compare the color distribution between paintings and graffiti. For this analysis, the bin size was 1 unit in CIELAB, i.e., colors are the same if they are less than 1 unit apart. Figure 9 shows the results of this analysis: panels A and B show the contour lines of the frequency of occurrence of the colors in paintings and graffiti, respectively; panels C and D represent the a* and b* histograms for paintings and graffiti, respectively. These data show that the colors in graffiti are used more isotopically than in paintings, i.e., the color distribution of graffiti is consistent with the fact that the corresponding gamut orientations are more spread out (Figure 9B,D), as also shown in Figure 8, while for paintings they are more pronounced on the b* axis (Figure 9A,C).

## 4. Discussion

We show here that graffiti artists and more traditional artists use colors in very similar ways. They produce similar colors in their compositions, expressed by the same balance and variability across perpendicular directions in the color space. In other words, the color gamuts of paintings and graffiti (see Figure 5) are similarly elongated (see the axis ratio in Figure 7 representing the balance) and oriented (see the angle in Figure 8 representing the direction of variability), showing a tendency for combining similar colors in the same proportions. Taken together, these facts suggest that, in terms of color structure, graffiti is comparable to paintings and thus is also an art expression.

There are, however, differences between graffiti and paintings. Graffiti display a greater diversity of colors, and the colors are more intense (see Figure 5 for the color gamut sizes of both graffiti and paintings). In other words, the colors are more saturated because graffiti artists are taking advantage of the new generation of artificial pigments available nowadays. Therefore, in graffiti, the gamut areas are larger (see Figure 6) and the orientations of the gamuts are more spread out, revealing a less tuned selection of the preferred colors (see Figure 8) and suggesting that graffiti artists have more freedom in color choices.

The chromatic structure of graffiti also carries regularities of the natural world. It was shown that paintings share a number of visual properties with natural scenes, particularly in terms of some color statistics [30]. Thus, it is probable that painters mimic, to a certain extent, the natural structure of the world, even in abstract artworks. Similarly to paintings, graffiti show a tendency to contain colors close to the yellow–blue axis (see Figure 5 and Figure 8), which are a characteristic of the natural environment [44,45], suggesting that graffiti artists’ color choices also mimic those of the natural world. In addition, the full color gamut of graffiti (see Figure 5) is very similar to that of natural scenes, even more than the traditional paintings [30], suggesting that graffiti is also bringing nature into the city.

This work is based on the analysis of a collection of graffiti from São Paulo, Brazil. This city is considered one of the major cities for street art [5] but still represents a limited sample. To estimate the extent to which these graffiti can be considered a representative sample of the graffiti production worldwide, we analyzed 434 photos download from google.com of graffiti from other major cities ranked as best graffiti cities in the world [46,47,48,49,50]: Berlin, Bogota, and Paris (listed in all five rankings); LA, London, Melbourne, and NYC (listed in four out of the five rankings). We analyzed their colors in the same way as described here but without colorimetric calibration. The results support the generalization claimed in this study as the same trends were revealed. A similar exercise was carried out with paintings. We selected 500 paintings of different epochs, painters and styles from a public database [51] and did the same analysis, also without colorimetric calibration. Again, the results were very similar, supporting the notion that our sample is representative of traditional paintings as well as of graffiti worldwide.

Graffiti have chromatic structures similar to those of paintings, revealing a tendency for combining similar colors in the same proportions. The preferred colors are also close to the same yellow–blue axis of color space, suggesting that, like more traditional artists, graffiti artists’ color choices mimic those of the natural environments. Even so, graffiti tend to have more saturated colors due to the availability of a new generation of synthetic pigments, resulting in a broader distribution of chromatic parameters than that of paintings and suggesting greater freedom in the choice of colors. In summary, by sharing their color structures with those of paintings, graffiti contribute to bringing art to the cities.
